# Supplementary material for: Antioxidant cysteine and methionine derivatives show trachea disruption in insects
Source: PLoS One. 2024 Oct 29;19(10):e0310919. doi: 10.1371/journal.pone.0310919 (PMC11521293; doi:10.1371/journal.pone.0310919)
Supplement: S1 Fig — (A) Female adult of R. pedestris. (B) Photograph of the whole midgut of R. pedestris. Abbreviations: M1, midgut first region; M2, midgut second region; M3, midgut third region; CR, constricted region; M4B, anterior bulb of midgut region; M4, midgut fourth region with crypts; H, hindgut. (C) Enlarged picture of the M4. (D) Developing trachea within the M4. The thin black tubes are trachea, observed here developing in the crypt. The asterisk represents the main duct passing through the middle of the M4. (PPTX) [file pone.0310919.s001.pptx]

## Slide 1
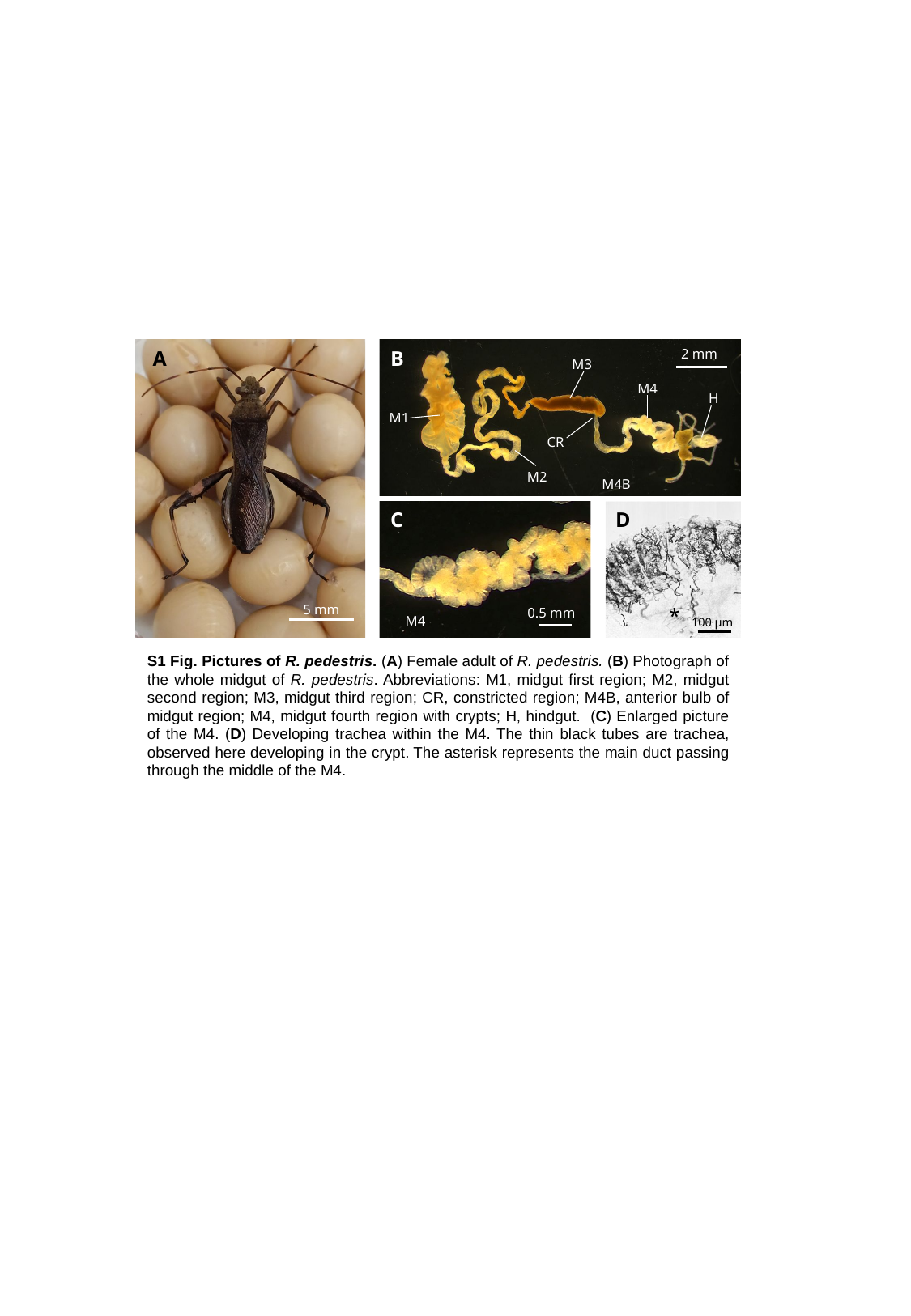

A
5 mm
B
2 mm
M3
M4
H
M1
CR
M2
M4B
D
*
100 µm
C
0.5 mm
M4
S1 Fig. Pictures of R. pedestris. (A) Female adult of R. pedestris. (B) Photograph of the whole midgut of R. pedestris. Abbreviations: M1, midgut first region; M2, midgut second region; M3, midgut third region; CR, constricted region; M4B, anterior bulb of midgut region; M4, midgut fourth region with crypts; H, hindgut. (C) Enlarged picture of the M4. (D) Developing trachea within the M4. The thin black tubes are trachea, observed here developing in the crypt. The asterisk represents the main duct passing through the middle of the M4.
