# Supplementary material for: Antioxidant cysteine and methionine derivatives show trachea disruption in insects
Source: PLoS One. 2024 Oct 29;19(10):e0310919. doi: 10.1371/journal.pone.0310919 (PMC11521293; doi:10.1371/journal.pone.0310919)
Supplement: S2 Fig — (PPTX) [file pone.0310919.s002.pptx]

## Slide 1
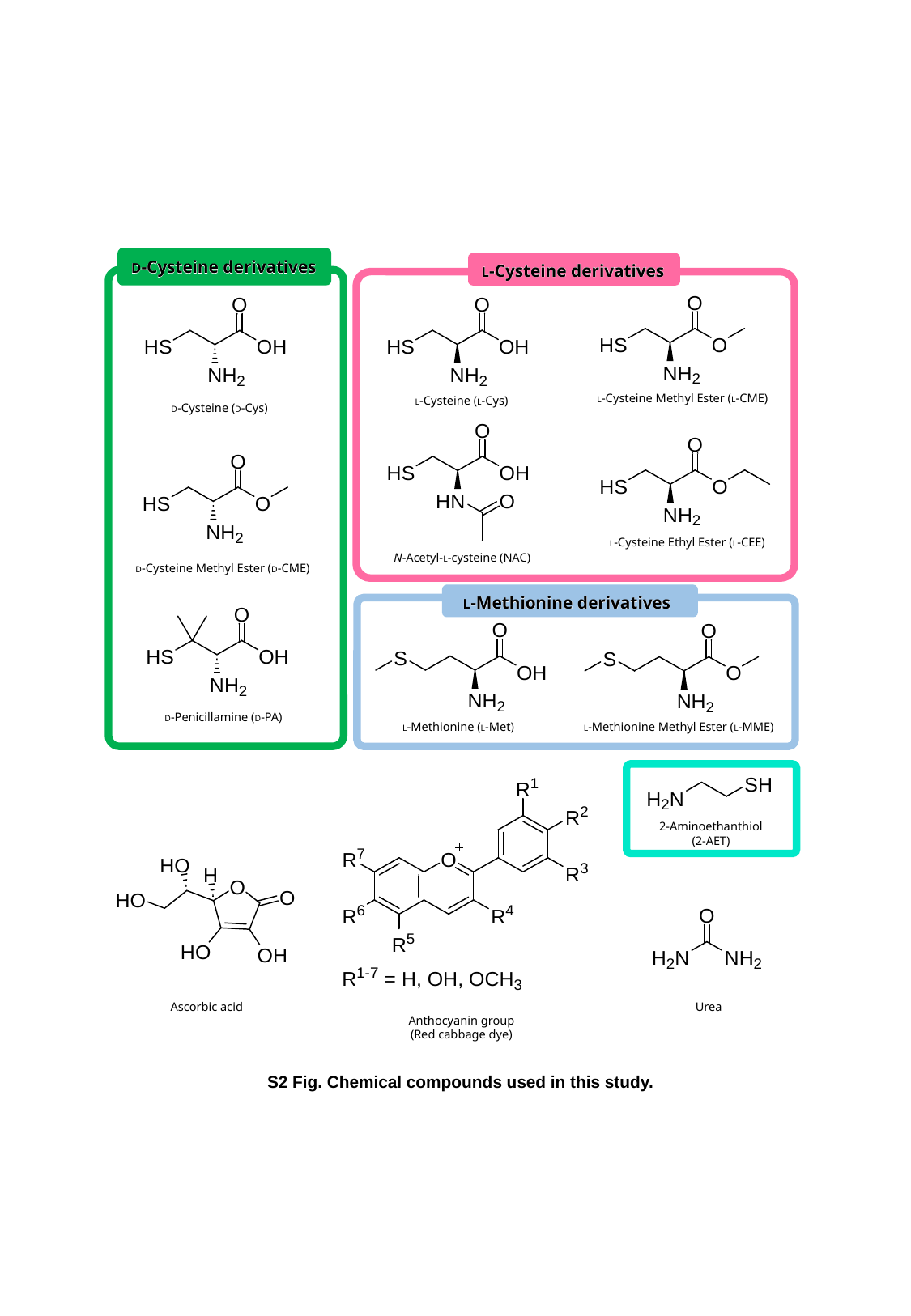

D-Cysteine derivatives
D-Cysteine derivatives
D-Cysteine (D-Cys)
D-Cysteine Methyl Ester (D-CME)
D-Penicillamine (D-PA)
L-Cysteine derivatives
L-Cysteine derivatives
L-Cysteine Methyl Ester (L-CME)
L-Cysteine (L-Cys)
L-Cysteine Ethyl Ester (L-CEE)
N-Acetyl-L-cysteine (NAC)
L-Methionine derivatives
L-Methionine derivatives
L-Methionine (L-Met)
L-Methionine Methyl Ester (L-MME)
2-Aminoethanthiol
(2-AET)
Ascorbic acid
Urea
Anthocyanin group
(Red cabbage dye)
S2 Fig. Chemical compounds used in this study.
