# Supplementary material for: Antioxidant cysteine and methionine derivatives show trachea disruption in insects
Source: PLoS One. 2024 Oct 29;19(10):e0310919. doi: 10.1371/journal.pone.0310919 (PMC11521293; doi:10.1371/journal.pone.0310919)
Supplement: S3 Fig — Survival rates and nymphal stages of R. pedestris. Observations started from the 3rd instar nymphs. The total numbers of insects at the starting time (day 0) are shown in brackets after the chemical names. (PPTX) [file pone.0310919.s003.pptx]

## Slide 1
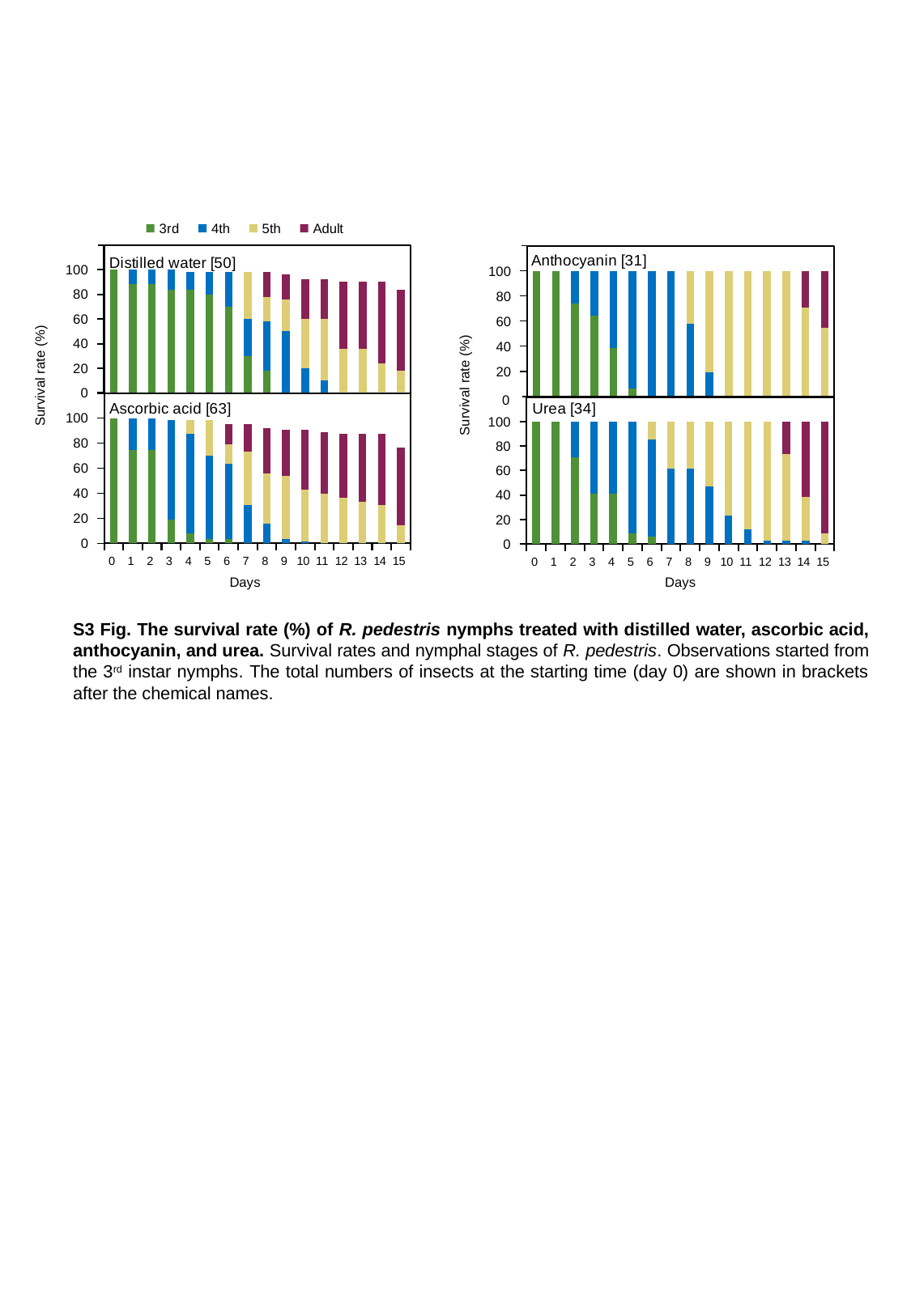

### Chart: Distilled water [50]
| Category | 2nd | 3rd | 4th | 5th | Adult |
|---|---|---|---|---|---|
| 0 | 0.0 | 100.0 | 0.0 | 0.0 | 0.0 |
| 1 | 0.0 | 88.0 | 12.0 | 0.0 | 0.0 |
| 2 | 0.0 | 88.0 | 12.0 | 0.0 | 0.0 |
| 3 | 0.0 | 84.0 | 16.0 | 0.0 | 0.0 |
| 4 | 0.0 | 84.0 | 14.000000000000002 | 0.0 | 0.0 |
| 5 | 0.0 | 80.0 | 18.0 | 0.0 | 0.0 |
| 6 | 0.0 | 70.0 | 28.000000000000004 | 0.0 | 0.0 |
| 7 | 0.0 | 30.0 | 30.0 | 38.0 | 0.0 |
| 8 | 0.0 | 18.0 | 40.0 | 20.0 | 20.0 |
| 9 | 0.0 | 0.0 | 50.0 | 26.0 | 20.0 |
| 10 | 0.0 | 0.0 | 20.0 | 40.0 | 32.0 |
| 11 | 0.0 | 0.0 | 10.0 | 50.0 | 32.0 |
| 12 | 0.0 | 0.0 | 0.0 | 36.0 | 54.0 |
| 13 | 0.0 | 0.0 | 0.0 | 36.0 | 54.0 |
| 14 | 0.0 | 0.0 | 0.0 | 24.0 | 66.0 |
| 15 | 0.0 | 0.0 | 0.0 | 18.0 | 66.0 |
Days
### Chart: Ascorbic acid [63]
| Category | 2nd | 3rd | 4th | 5th | Adult |
|---|---|---|---|---|---|
| 0 | 0.0 | 100.0 | 0.0 | 0.0 | 0.0 |
| 1 | 0.0 | 74.60317460317461 | 25.396825396825395 | 0.0 | 0.0 |
| 2 | 0.0 | 74.60317460317461 | 25.396825396825395 | 0.0 | 0.0 |
| 3 | 0.0 | 19.047619047619047 | 79.36507936507937 | 0.0 | 0.0 |
| 4 | 0.0 | 7.936507936507936 | 79.36507936507937 | 11.11111111111111 | 0.0 |
| 5 | 0.0 | 3.1746031746031744 | 66.66666666666666 | 28.57142857142857 | 0.0 |
| 6 | 0.0 | 3.1746031746031744 | 60.317460317460316 | 15.873015873015872 | 15.873015873015872 |
| 7 | 0.0 | 0.0 | 30.158730158730158 | 42.857142857142854 | 22.22222222222222 |
| 8 | 0.0 | 0.0 | 15.873015873015872 | 39.682539682539684 | 36.507936507936506 |
| 9 | 0.0 | 0.0 | 3.1746031746031744 | 50.79365079365079 | 36.507936507936506 |
| 10 | 0.0 | 0.0 | 1.5873015873015872 | 41.269841269841265 | 47.61904761904761 |
| 11 | 0.0 | 0.0 | 0.0 | 39.682539682539684 | 49.2063492063492 |
| 12 | 0.0 | 0.0 | 0.0 | 36.507936507936506 | 50.79365079365079 |
| 13 | 0.0 | 0.0 | 0.0 | 33.33333333333333 | 53.96825396825397 |
| 14 | 0.0 | 0.0 | 0.0 | 30.158730158730158 | 57.14285714285714 |
| 15 | 0.0 | 0.0 | 0.0 | 14.285714285714285 | 61.904761904761905 |
### Chart: Anthocyanin [31]
| Category | 2nd | 3rd | 4th | 5th | Adult |
|---|---|---|---|---|---|
| 0 | 0.0 | 100.0 | 0.0 | 0.0 | 0.0 |
| 1 | 0.0 | 100.0 | 0.0 | 0.0 | 0.0 |
| 2 | 0.0 | 74.19354838709677 | 25.806451612903224 | 0.0 | 0.0 |
| 3 | 0.0 | 64.51612903225806 | 35.483870967741936 | 0.0 | 0.0 |
| 4 | 0.0 | 38.70967741935484 | 61.29032258064516 | 0.0 | 0.0 |
| 5 | 0.0 | 6.451612903225806 | 93.54838709677419 | 0.0 | 0.0 |
| 6 | 0.0 | 0.0 | 100.0 | 0.0 | 0.0 |
| 7 | 0.0 | 0.0 | 100.0 | 0.0 | 0.0 |
| 8 | 0.0 | 0.0 | 58.06451612903226 | 41.935483870967744 | 0.0 |
| 9 | 0.0 | 0.0 | 19.35483870967742 | 80.64516129032258 | 0.0 |
| 10 | 0.0 | 0.0 | 0.0 | 100.0 | 0.0 |
| 11 | 0.0 | 0.0 | 0.0 | 100.0 | 0.0 |
| 12 | 0.0 | 0.0 | 0.0 | 100.0 | 0.0 |
| 13 | 0.0 | 0.0 | 0.0 | 100.0 | 0.0 |
| 14 | 0.0 | 0.0 | 0.0 | 70.96774193548387 | 29.03225806451613 |
| 15 | 0.0 | 0.0 | 0.0 | 54.83870967741935 | 45.16129032258064 |
### Chart: Urea [34]
| Category | 2nd | 3rd | 4th | 5th | Adult |
|---|---|---|---|---|---|
| 0 | 0.0 | 100.0 | 0.0 | 0.0 | 0.0 |
| 1 | 0.0 | 100.0 | 0.0 | 0.0 | 0.0 |
| 2 | 0.0 | 70.58823529411765 | 29.411764705882355 | 0.0 | 0.0 |
| 3 | 0.0 | 41.17647058823529 | 58.82352941176471 | 0.0 | 0.0 |
| 4 | 0.0 | 41.17647058823529 | 58.82352941176471 | 0.0 | 0.0 |
| 5 | 0.0 | 8.823529411764707 | 91.17647058823529 | 0.0 | 0.0 |
| 6 | 0.0 | 5.88235294117647 | 79.41176470588235 | 14.705882352941178 | 0.0 |
| 7 | 0.0 | 0.0 | 61.76470588235294 | 38.23529411764706 | 0.0 |
| 8 | 0.0 | 0.0 | 61.76470588235294 | 38.23529411764706 | 0.0 |
| 9 | 0.0 | 0.0 | 47.05882352941176 | 52.94117647058824 | 0.0 |
| 10 | 0.0 | 0.0 | 23.52941176470588 | 76.47058823529412 | 0.0 |
| 11 | 0.0 | 0.0 | 11.76470588235294 | 88.23529411764706 | 0.0 |
| 12 | 0.0 | 0.0 | 2.941176470588235 | 97.05882352941177 | 0.0 |
| 13 | 0.0 | 0.0 | 2.941176470588235 | 70.58823529411765 | 26.47058823529412 |
| 14 | 0.0 | 0.0 | 2.941176470588235 | 35.294117647058826 | 61.76470588235294 |
| 15 | 0.0 | 0.0 | 0.0 | 8.823529411764707 | 91.17647058823529 |Days
0
Survival rate (%)
Survival rate (%)
S3 Fig. The survival rate (%) of R. pedestris nymphs treated with distilled water, ascorbic acid, anthocyanin, and urea. Survival rates and nymphal stages of R. pedestris. Observations started from the 3rd instar nymphs. The total numbers of insects at the starting time (day 0) are shown in brackets after the chemical names.
