# Supplementary material for: Antioxidant cysteine and methionine derivatives show trachea disruption in insects
Source: PLoS One. 2024 Oct 29;19(10):e0310919. doi: 10.1371/journal.pone.0310919 (PMC11521293; doi:10.1371/journal.pone.0310919)
Supplement: S4 Fig — Observations started from the 3rd instar nymphs. Color indicates developmental stage: green, 3rd instar; blue, 4th instar; yellow, 5th instar; brown, adult. Red Arrows indicate when all individuals died. The total numbers of insects at the starting time (day 0) are shown in brackets after the chemical names. Abbreviations: NAC, N-Acetyl-L-cysteine; L-Cys, L-cysteine; L-CME, L-cysteine methyl ester hydrochloride; L-CEE, L-cysteine ethyl ester hydrochloride; D-Cys, D-cysteine; D-CME, D-cysteine methyl ester hydrochloride; D-PA, D-penicillamine; 2-AET, 2-amino ethanethiol; L-Met, L-methionine; L-MME, L-methionine methyl ester hydrochloride. (PPTX) [file pone.0310919.s004.pptx]

## Slide 1
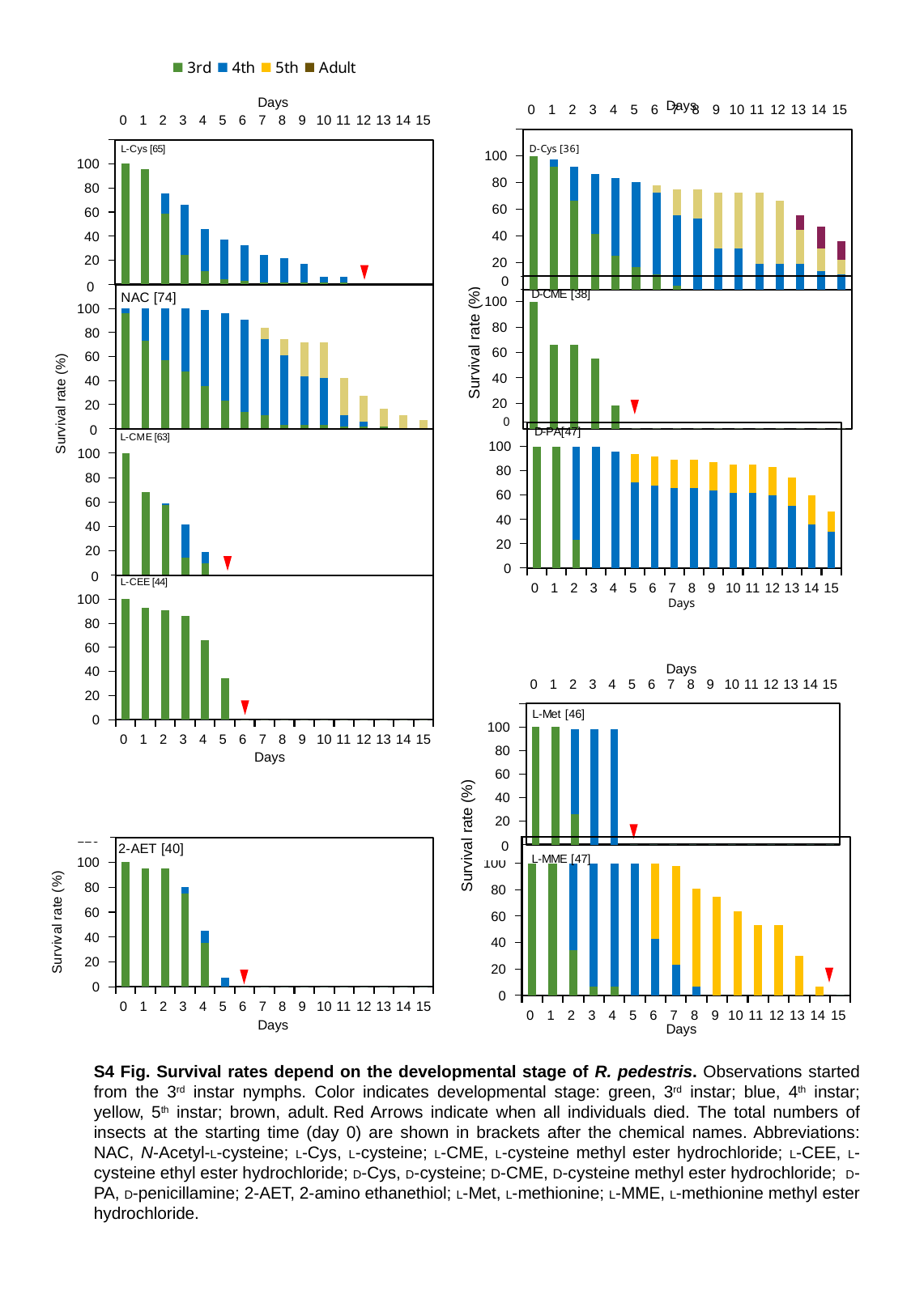

### Chart: L-Cys [65]
| Category | 2nd | 3rd | 4th | 5th | Adult |
|---|---|---|---|---|---|
| 0 | 0.0 | 100.0 | 0.0 | 0.0 | 0.0 |
| 1 | 0.0 | 95.38461538461539 | 0.0 | 0.0 | 0.0 |
| 2 | 0.0 | 58.46153846153847 | 16.923076923076923 | 0.0 | 0.0 |
| 3 | 0.0 | 24.615384615384617 | 41.53846153846154 | 0.0 | 0.0 |
| 4 | 0.0 | 10.76923076923077 | 35.38461538461539 | 0.0 | 0.0 |
| 5 | 0.0 | 4.615384615384616 | 32.30769230769231 | 0.0 | 0.0 |
| 6 | 0.0 | 3.076923076923077 | 29.230769230769234 | 0.0 | 0.0 |
| 7 | 0.0 | 1.5384615384615385 | 23.076923076923077 | 0.0 | 0.0 |
| 8 | 0.0 | 1.5384615384615385 | 20.0 | 0.0 | 0.0 |
| 9 | 0.0 | 1.5384615384615385 | 15.384615384615385 | 0.0 | 0.0 |
| 10 | 0.0 | 1.5384615384615385 | 4.615384615384616 | 0.0 | 0.0 |
| 11 | 0.0 | 1.5384615384615385 | 4.615384615384616 | 0.0 | 0.0 |
| 12 | 0.0 | 0.0 | 0.0 | 0.0 | 0.0 |
| 13 | 0.0 | 0.0 | 0.0 | 0.0 | 0.0 |
| 14 | 0.0 | 0.0 | 0.0 | 0.0 | 0.0 |
| 15 | 0.0 | 0.0 | 0.0 | 0.0 | 0.0 |Days
### Chart: NAC [74]
| Category | 2nd | 3rd | 4th | 5th | Adult |
|---|---|---|---|---|---|
| 0 | 0.0 | 95.94594594594594 | 4.054054054054054 | 0.0 | 0.0 |
| 1 | 0.0 | 72.97297297297297 | 27.027027027027028 | 0.0 | 0.0 |
| 2 | 0.0 | 56.75675675675676 | 43.24324324324324 | 0.0 | 0.0 |
| 3 | 0.0 | 47.2972972972973 | 52.702702702702695 | 0.0 | 0.0 |
| 4 | 0.0 | 35.13513513513514 | 63.51351351351351 | 0.0 | 0.0 |
| 5 | 0.0 | 22.972972972972975 | 72.97297297297297 | 0.0 | 0.0 |
| 6 | 0.0 | 13.513513513513514 | 77.02702702702703 | 0.0 | 0.0 |
| 7 | 0.0 | 10.81081081081081 | 63.51351351351351 | 9.45945945945946 | 0.0 |
| 8 | 0.0 | 2.7027027027027026 | 58.108108108108105 | 13.513513513513514 | 0.0 |
| 9 | 0.0 | 2.7027027027027026 | 40.54054054054054 | 28.37837837837838 | 0.0 |
| 10 | 0.0 | 2.7027027027027026 | 39.189189189189186 | 29.72972972972973 | 0.0 |
| 11 | 0.0 | 1.3513513513513513 | 9.45945945945946 | 31.08108108108108 | 0.0 |
| 12 | 0.0 | 1.3513513513513513 | 4.054054054054054 | 21.62162162162162 | 0.0 |
| 13 | 0.0 | 1.3513513513513513 | 0.0 | 14.864864864864865 | 0.0 |
| 14 | 0.0 | 0.0 | 0.0 | 10.81081081081081 | 0.0 |
| 15 | 0.0 | 0.0 | 0.0 | 6.756756756756757 | 0.0 |
0
### Chart: L-CME [63]
| Category | 2nd | 3rd | 4th | 5th | Adult |
|---|---|---|---|---|---|
| 0 | 0.0 | 100.0 | 0.0 | 0.0 | 0.0 |
| 1 | 0.0 | 68.25396825396825 | 0.0 | 0.0 | 0.0 |
| 2 | 0.0 | 57.14285714285714 | 1.5873015873015872 | 0.0 | 0.0 |
| 3 | 0.0 | 14.285714285714285 | 26.984126984126984 | 0.0 | 0.0 |
| 4 | 0.0 | 9.523809523809524 | 9.523809523809524 | 0.0 | 0.0 |
| 5 | 0.0 | 0.0 | 0.0 | 0.0 | 0.0 |
| 6 | 0.0 | 0.0 | 0.0 | 0.0 | 0.0 |
| 7 | 0.0 | 0.0 | 0.0 | 0.0 | 0.0 |
| 8 | 0.0 | 0.0 | 0.0 | 0.0 | 0.0 |
| 9 | 0.0 | 0.0 | 0.0 | 0.0 | 0.0 |
| 10 | 0.0 | 0.0 | 0.0 | 0.0 | 0.0 |
| 11 | 0.0 | 0.0 | 0.0 | 0.0 | 0.0 |
| 12 | 0.0 | 0.0 | 0.0 | 0.0 | 0.0 |
| 13 | 0.0 | 0.0 | 0.0 | 0.0 | 0.0 |
| 14 | 0.0 | 0.0 | 0.0 | 0.0 | 0.0 |
| 15 | 0.0 | 0.0 | 0.0 | 0.0 | 0.0 |Survival rate (%)
0
### Chart: L-CEE [44]
| Category | 2nd | 3rd | 4th | 5th | Adult |
|---|---|---|---|---|---|
| 0 | 0.0 | 100.0 | 0.0 | 0.0 | 0.0 |
| 1 | 0.0 | 93.18181818181817 | 0.0 | 0.0 | 0.0 |
| 2 | 0.0 | 90.9090909090909 | 0.0 | 0.0 | 0.0 |
| 3 | 0.0 | 86.36363636363636 | 0.0 | 0.0 | 0.0 |
| 4 | 0.0 | 65.9090909090909 | 0.0 | 0.0 | 0.0 |
| 5 | 0.0 | 34.090909090909086 | 0.0 | 0.0 | 0.0 |
| 6 | 0.0 | 0.0 | 0.0 | 0.0 | 0.0 |
| 7 | 0.0 | 0.0 | 0.0 | 0.0 | 0.0 |
| 8 | 0.0 | 0.0 | 0.0 | 0.0 | 0.0 |
| 9 | 0.0 | 0.0 | 0.0 | 0.0 | 0.0 |
| 10 | 0.0 | 0.0 | 0.0 | 0.0 | 0.0 |
| 11 | 0.0 | 0.0 | 0.0 | 0.0 | 0.0 |
| 12 | 0.0 | 0.0 | 0.0 | 0.0 | 0.0 |
| 13 | 0.0 | 0.0 | 0.0 | 0.0 | 0.0 |
| 14 | 0.0 | 0.0 | 0.0 | 0.0 | 0.0 |
| 15 | 0.0 | 0.0 | 0.0 | 0.0 | 0.0 |
0
Days
### Chart: D-Cys [36]
| Category | 2nd | 3rd | 4th | 5th | Adult |
|---|---|---|---|---|---|
| 0 | 0.0 | 100.0 | 0.0 | 0.0 | 0.0 |
| 1 | 0.0 | 91.66666666666666 | 5.555555555555555 | 0.0 | 0.0 |
| 2 | 0.0 | 66.66666666666666 | 25.0 | 0.0 | 0.0 |
| 3 | 0.0 | 41.66666666666667 | 44.44444444444444 | 0.0 | 0.0 |
| 4 | 0.0 | 25.0 | 58.333333333333336 | 0.0 | 0.0 |
| 5 | 0.0 | 16.666666666666664 | 63.888888888888886 | 0.0 | 0.0 |
| 6 | 0.0 | 11.11111111111111 | 61.111111111111114 | 5.555555555555555 | 0.0 |
| 7 | 0.0 | 2.7777777777777777 | 52.77777777777778 | 19.444444444444446 | 0.0 |
| 8 | 0.0 | 0.0 | 52.77777777777778 | 22.22222222222222 | 0.0 |
| 9 | 0.0 | 0.0 | 30.555555555555557 | 41.66666666666667 | 0.0 |
| 10 | 0.0 | 0.0 | 30.555555555555557 | 41.66666666666667 | 0.0 |
| 11 | 0.0 | 0.0 | 19.444444444444446 | 52.77777777777778 | 0.0 |
| 12 | 0.0 | 0.0 | 19.444444444444446 | 47.22222222222222 | 0.0 |
| 13 | 0.0 | 0.0 | 19.444444444444446 | 25.0 | 11.11111111111111 |
| 14 | 0.0 | 0.0 | 13.88888888888889 | 16.666666666666664 | 16.666666666666664 |
| 15 | 0.0 | 0.0 | 11.11111111111111 | 11.11111111111111 | 13.88888888888889 |Days
### Chart: D-CME [38]
| Category | 2nd | 3rd | 4th | 5th | Adult |
|---|---|---|---|---|---|
| 0 | 0.0 | 100.0 | 0.0 | 0.0 | 0.0 |
| 1 | 0.0 | 65.78947368421053 | 0.0 | 0.0 | 0.0 |
| 2 | 0.0 | 65.78947368421053 | 0.0 | 0.0 | 0.0 |
| 3 | 0.0 | 55.26315789473685 | 0.0 | 0.0 | 0.0 |
| 4 | 0.0 | 18.421052631578945 | 0.0 | 0.0 | 0.0 |
| 5 | 0.0 | 0.0 | 0.0 | 0.0 | 0.0 |
| 6 | 0.0 | 0.0 | 0.0 | 0.0 | 0.0 |
| 7 | 0.0 | 0.0 | 0.0 | 0.0 | 0.0 |
| 8 | 0.0 | 0.0 | 0.0 | 0.0 | 0.0 |
| 9 | 0.0 | 0.0 | 0.0 | 0.0 | 0.0 |
| 10 | 0.0 | 0.0 | 0.0 | 0.0 | 0.0 |
| 11 | 0.0 | 0.0 | 0.0 | 0.0 | 0.0 |
| 12 | 0.0 | 0.0 | 0.0 | 0.0 | 0.0 |
| 13 | 0.0 | 0.0 | 0.0 | 0.0 | 0.0 |
| 14 | 0.0 | 0.0 | 0.0 | 0.0 | 0.0 |
| 15 | 0.0 | 0.0 | 0.0 | 0.0 | 0.0 |0
Survival rate (%)
0
Days
### Chart: D-PA[47]
| Category | 2nd | 3rd | 4th | 5th | Adult |
|---|---|---|---|---|---|
| 0 | 0.0 | 100.0 | 0.0 | 0.0 | 0.0 |
| 1 | 0.0 | 100.0 | 0.0 | 0.0 | 0.0 |
| 2 | 0.0 | 23.404255319148938 | 76.59574468085107 | 0.0 | 0.0 |
| 3 | 0.0 | 0.0 | 100.0 | 0.0 | 0.0 |
| 4 | 0.0 | 0.0 | 95.74468085106383 | 0.0 | 0.0 |
| 5 | 0.0 | 0.0 | 70.2127659574468 | 23.404255319148938 | 0.0 |
| 6 | 0.0 | 0.0 | 68.08510638297872 | 23.404255319148938 | 0.0 |
| 7 | 0.0 | 0.0 | 65.95744680851064 | 23.404255319148938 | 0.0 |
| 8 | 0.0 | 0.0 | 65.95744680851064 | 23.404255319148938 | 0.0 |
| 9 | 0.0 | 0.0 | 63.829787234042556 | 23.404255319148938 | 0.0 |
| 10 | 0.0 | 0.0 | 61.702127659574465 | 23.404255319148938 | 0.0 |
| 11 | 0.0 | 0.0 | 61.702127659574465 | 23.404255319148938 | 0.0 |
| 12 | 0.0 | 0.0 | 59.57446808510638 | 23.404255319148938 | 0.0 |
| 13 | 0.0 | 0.0 | 51.06382978723404 | 23.404255319148938 | 0.0 |
| 14 | 0.0 | 0.0 | 36.17021276595745 | 23.404255319148938 | 0.0 |
| 15 | 0.0 | 0.0 | 29.78723404255319 | 17.02127659574468 | 0.0 |
0
### Chart: L-Met [46]
| Category | 2nd | 3rd | 4th | 5th | Adult |
|---|---|---|---|---|---|
| 0 | 0.0 | 100.0 | 0.0 | 0.0 | 0.0 |
| 1 | 0.0 | 100.0 | 0.0 | 0.0 | 0.0 |
| 2 | 0.0 | 26.08695652173913 | 71.73913043478261 | 0.0 | 0.0 |
| 3 | 0.0 | 0.0 | 97.82608695652173 | 0.0 | 0.0 |
| 4 | 0.0 | 0.0 | 97.82608695652173 | 0.0 | 0.0 |
| 5 | 0.0 | 0.0 | 0.0 | 0.0 | 0.0 |
| 6 | 0.0 | 0.0 | 0.0 | 0.0 | 0.0 |
| 7 | 0.0 | 0.0 | 0.0 | 0.0 | 0.0 |
| 8 | 0.0 | 0.0 | 0.0 | 0.0 | 0.0 |
| 9 | 0.0 | 0.0 | 0.0 | 0.0 | 0.0 |
| 10 | 0.0 | 0.0 | 0.0 | 0.0 | 0.0 |
| 11 | 0.0 | 0.0 | 0.0 | 0.0 | 0.0 |
| 12 | 0.0 | 0.0 | 0.0 | 0.0 | 0.0 |
| 13 | 0.0 | 0.0 | 0.0 | 0.0 | 0.0 |
| 14 | 0.0 | 0.0 | 0.0 | 0.0 | 0.0 |
| 15 | 0.0 | 0.0 | 0.0 | 0.0 | 0.0 |Days
Survival rate (%)
0
Days
### Chart: L-MME [47]
| Category | 2nd | 3rd | 4th | 5th | Adult |
|---|---|---|---|---|---|
| 0 | 0.0 | 100.0 | 0.0 | 0.0 | 0.0 |
| 1 | 0.0 | 100.0 | 0.0 | 0.0 | 0.0 |
| 2 | 0.0 | 34.04255319148936 | 65.95744680851064 | 0.0 | 0.0 |
| 3 | 0.0 | 6.382978723404255 | 93.61702127659575 | 0.0 | 0.0 |
| 4 | 0.0 | 6.382978723404255 | 93.61702127659575 | 0.0 | 0.0 |
| 5 | 0.0 | 0.0 | 100.0 | 0.0 | 0.0 |
| 6 | 0.0 | 0.0 | 42.5531914893617 | 57.446808510638306 | 0.0 |
| 7 | 0.0 | 0.0 | 23.404255319148938 | 74.46808510638297 | 0.0 |
| 8 | 0.0 | 0.0 | 6.382978723404255 | 74.46808510638297 | 0.0 |
| 9 | 0.0 | 0.0 | 0.0 | 74.46808510638297 | 0.0 |
| 10 | 0.0 | 0.0 | 0.0 | 63.829787234042556 | 0.0 |
| 11 | 0.0 | 0.0 | 0.0 | 53.191489361702125 | 0.0 |
| 12 | 0.0 | 0.0 | 0.0 | 53.191489361702125 | 0.0 |
| 13 | 0.0 | 0.0 | 0.0 | 29.78723404255319 | 0.0 |
| 14 | 0.0 | 0.0 | 0.0 | 6.382978723404255 | 0.0 |
| 15 | 0.0 | 0.0 | 0.0 | 0.0 | 0.0 |
0
### Chart: 2-AET [40]
| Category | 2nd | 3rd | 4th | 5th | Adult |
|---|---|---|---|---|---|
| 0 | 0.0 | 100.0 | 0.0 | 0.0 | 0.0 |
| 1 | 0.0 | 95.0 | 0.0 | 0.0 | 0.0 |
| 2 | 0.0 | 95.0 | 0.0 | 0.0 | 0.0 |
| 3 | 0.0 | 75.0 | 5.0 | 0.0 | 0.0 |
| 4 | 0.0 | 35.0 | 10.0 | 0.0 | 0.0 |
| 5 | 0.0 | 0.0 | 7.5 | 0.0 | 0.0 |
| 6 | 0.0 | 0.0 | 0.0 | 0.0 | 0.0 |
| 7 | 0.0 | 0.0 | 0.0 | 0.0 | 0.0 |
| 8 | 0.0 | 0.0 | 0.0 | 0.0 | 0.0 |
| 9 | 0.0 | 0.0 | 0.0 | 0.0 | 0.0 |
| 10 | 0.0 | 0.0 | 0.0 | 0.0 | 0.0 |
| 11 | 0.0 | 0.0 | 0.0 | 0.0 | 0.0 |
| 12 | 0.0 | 0.0 | 0.0 | 0.0 | 0.0 |
| 13 | 0.0 | 0.0 | 0.0 | 0.0 | 0.0 |
| 14 | 0.0 | 0.0 | 0.0 | 0.0 | 0.0 |
| 15 | 0.0 | 0.0 | 0.0 | 0.0 | 0.0 |
Days
S4 Fig. Survival rates depend on the developmental stage of R. pedestris. Observations started from the 3rd instar nymphs. Color indicates developmental stage: green, 3rd instar; blue, 4th instar; yellow, 5th instar; brown, adult. Red Arrows indicate when all individuals died. The total numbers of insects at the starting time (day 0) are shown in brackets after the chemical names. Abbreviations: NAC, N-Acetyl-L-cysteine; L-Cys, L-cysteine; L-CME, L-cysteine methyl ester hydrochloride; L-CEE, L-cysteine ethyl ester hydrochloride; D-Cys, D-cysteine; D-CME, D-cysteine methyl ester hydrochloride; D-PA, D-penicillamine; 2-AET, 2-amino ethanethiol; L-Met, L-methionine; L-MME, L-methionine methyl ester hydrochloride.
