# Supplementary material for: Antioxidant cysteine and methionine derivatives show trachea disruption in insects
Source: PLoS One. 2024 Oct 29;19(10):e0310919. doi: 10.1371/journal.pone.0310919 (PMC11521293; doi:10.1371/journal.pone.0310919)
Supplement: S5 Fig — Results of female (F) and male (M) are shown. Mortalities were observed 7 days after treatment (mean ± SD, n = 3). In each replicate, five female and five male individuals were reared in a petri dish, and their mortality was observed. Abbreviations: DW, Distilled water; NAC, N-Acetyl-L-cysteine; L-Cys, L-cysteine; L-CME, L-cysteine methyl ester hydrochloride; L-CEE, L-cysteine ethyl ester hydrochloride; D-Cys, D-cysteine; D-CME, D-cysteine methyl ester hydrochloride; D-PA, D-penicillamine; 2-AET, 2-amino ethanethiol; L-Met, L-methionine; L-MME, L-methionine methyl ester hydrochloride. The significant differences were determined by the Mann-Whitney U test (p < 0.05) between females and males. n.s.: not significant. (PPTX) [file pone.0310919.s005.pptx]

## Slide 1
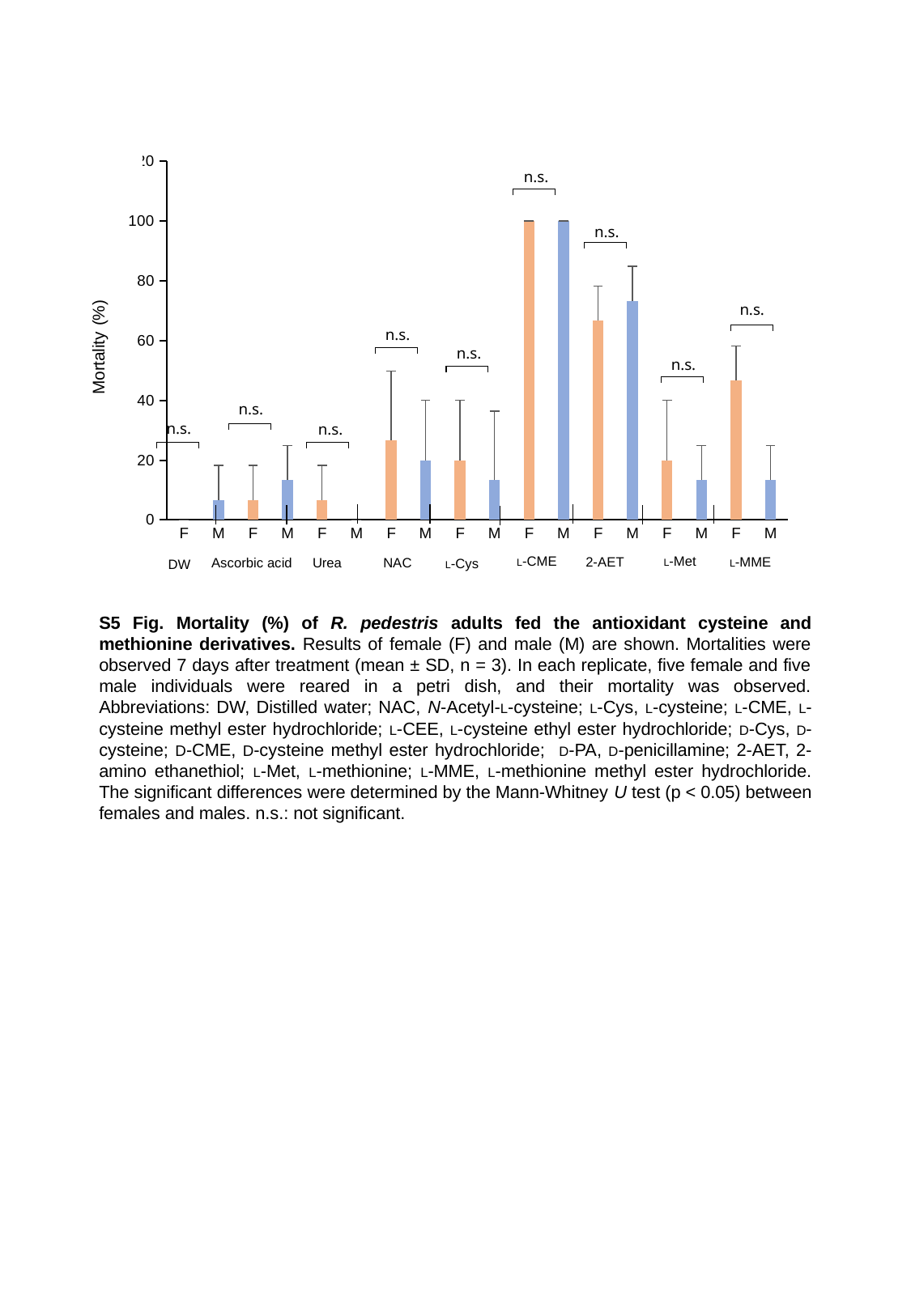

### Chart
| Category | Average_of_mortality |
|---|---|
| F | 0.0 |
| M | 6.666666666666667 |
| F | 6.666666666666667 |
| M | 13.333333333333334 |
| F | 6.666666666666667 |
| M | 0.0 |
| F | 26.666666666666668 |
| M | 20.0 |
| F | 20.0 |
| M | 13.333333333333334 |
| F | 100.0 |
| M | 100.0 |
| F | 66.66666666666667 |
| M | 73.33333333333333 |
| F | 20.0 |
| M | 13.333333333333334 |
| F | 46.666666666666664 |
| M | 13.333333333333334 |n.s.
n.s.
n.s.
n.s.
n.s.
n.s.
n.s.
n.s.
n.s.
L-CME
L-Met
L-MME
2-AET
Urea
Ascorbic acid
NAC
L-Cys
DW
S5 Fig. Mortality (%) of R. pedestris adults fed the antioxidant cysteine and methionine derivatives. Results of female (F) and male (M) are shown. Mortalities were observed 7 days after treatment (mean ± SD, n = 3). In each replicate, five female and five male individuals were reared in a petri dish, and their mortality was observed. Abbreviations: DW, Distilled water; NAC, N-Acetyl-L-cysteine; L-Cys, L-cysteine; L-CME, L-cysteine methyl ester hydrochloride; L-CEE, L-cysteine ethyl ester hydrochloride; D-Cys, D-cysteine; D-CME, D-cysteine methyl ester hydrochloride; D-PA, D-penicillamine; 2-AET, 2-amino ethanethiol; L-Met, L-methionine; L-MME, L-methionine methyl ester hydrochloride. The significant differences were determined by the Mann-Whitney U test (p < 0.05) between females and males. n.s.: not significant.
