# Supplementary material for: Antioxidant cysteine and methionine derivatives show trachea disruption in insects
Source: PLoS One. 2024 Oct 29;19(10):e0310919. doi: 10.1371/journal.pone.0310919 (PMC11521293; doi:10.1371/journal.pone.0310919)
Supplement: S1 Table — (PDF) [file pone.0310919.s006.pdf]

Supplementary Table 1 Details of DTN area in 4th instar of *R. pedestris* .

Number of insect[a]	Number of Picture[b]	Area[c]								
		Treatments[d]								
		**DW**	**Acorbic acid**	**Urea**	**NAC**	**L-Cys**	**L-CME**	**2-AET**	**L-Met**	**L-MME**
1	1	4.09	4.50	3.17	1.10	0.97	1.56	1.08	1.86	3.29
1	2	4.09	6.58	5.63	1.24	2.08	0.96	1.09	2.99	2.90
1	3	4.88	6.53	4.59	1.56	1.52	0.80	0.99	2.09	2.80
2	1	4.82	4.72	4.17	1.73	1.66	0.62	0.99	3.11	1.51
2	2	5.62	6.63	5.32	1.76	1.21	0.98	0.87	2.56	1.90
2	3	5.43	6.13	5.42	1.03	1.21	1.21	0.98	2.99	3.99
3	1	6.46	3.87	6.82	0.97	1.56	1.06	0.97	1.77	1.80
3	2	6.34	4.60	5.67	0.87	1.45	0.84	1.10	1.90	1.88
3	3	4.57	4.99	6.52	1.45	0.93	0.90	0.87	3.09	3.10
4	1	4.88	4.46	4.65	1.35	2.70	1.24	0.85	1.65	2.65
4	2	4.45	4.60	5.68	1.63	1.10	0.99	1.13	2.10	2.78
4	3	5.65	4.90	5.44	1.10	0.98	0.84	1.40	2.56	1.59
5	1	5.49	4.23	4.99	1.45	1.08	1.07	2.46	1.87	2.74
5	2	6.88	6.76	6.75	1.39	1.98	1.45	1.30	3.10	3.09
5	3	6.02	6.15	4.53	1.07	0.88	1.04	0.88	2.77	3.90

[a] 5 individuals of 4th instars were used for this experiment. The pictures were taken 7 days after treatment.

[b] The 3 photos of a part of each symbiotic organ in a 4th instar were taken by laser confocal microscope.

[c] The area means mm^2. ImageJ software (https://imagej.net/ij/) measured the DTN area (mm^2/insect) with a total area of 3 pictures.

[d] Each abbreviation means the following: DW, Distilled water; NAC, *N* -Acetyl-L-cysteine; L-Cys, L-cysteine; L-CME, L-cysteine methyl ester hydrochloride; 2-AET, 2-amino ethanethiol; L-Met, L-methionine; L-MME, L-methionine methyl ester hydrochloride.
