# Supplementary material for: Antioxidant cysteine and methionine derivatives show trachea disruption in insects
Source: PLoS One. 2024 Oct 29;19(10):e0310919. doi: 10.1371/journal.pone.0310919 (PMC11521293; doi:10.1371/journal.pone.0310919)
Supplement: S2 Table — (PDF) [file pone.0310919.s007.pdf]

Supplementary Table 2 Details of DTN area in adults of *R. pedestris* .

Number of insect[a]	Number of Picture[b]	Area[c]								
		Treatments[d]								
		DW	Acorbic acid	Urea	NAC	L-Cys	L-CME	2-AET	L-Met	L-MME
1	1	7.05	5.50	8.59	1.79	1.73	0.97	0.17	1.52	0.98
1	2	8.12	5.63	7.76	0.75	1.22	1.36	0.25	1.03	1.26
1	3	7.76	9.85	6.73	1.21	0.67	0.74	0.51	1.68	1.88
1	4	6.91	7.60	5.13	1.45	1.36	0.48	0.38	1.54	2.01
1	5	7.76	7.17	5.37	0.70	0.85	0.50	0.42	1.61	0.47
2	1	6.75	6.75	5.64	0.72	0.45	0.67	0.46	1.06	1.10
2	2	5.57	6.49	6.53	1.51	1.50	0.75	0.35	1.02	0.87
2	3	6.81	8.82	5.47	1.08	0.56	0.78	0.45	1.65	0.98
2	4	6.25	5.61	8.93	0.56	1.53	0.87	0.90	1.41	0.89
2	5	6.55	6.80	8.00	0.80	1.19	0.50	0.68	1.58	1.30
3	1	5.78	8.89	6.73	0.79	1.76	0.78	0.18	1.71	0.99
3	2	6.72	5.83	6.68	0.78	0.35	0.88	0.25	1.60	0.97
3	3	6.62	6.74	6.90	0.87	1.56	0.99	0.37	1.58	0.67
3	4	7.71	7.65	7.09	1.15	0.47	0.76	0.47	1.98	1.28
3	5	7.80	5.60	5.17	0.45	0.41	0.98	0.50	1.46	0.92
4	1	5.90	5.99	6.20	1.79	1.21	0.32	0.47	1.02	0.65
4	2	6.69	7.77	8.52	0.87	0.99	1.00	1.00	1.29	1.03
4	3	7.90	7.73	8.71	0.99	0.91	0.87	0.77	1.08	1.42
4	4	7.87	5.63	5.67	1.10	0.65	0.86	0.65	1.03	1.02
4	5	5.01	6.01	5.62	0.78	1.67	0.72	1.00	1.04	0.97
5	1	7.83	5.08	6.73	0.96	0.87	0.76	0.54	1.71	0.86
5	2	7.62	7.11	7.32	0.86	0.76	0.33	0.60	1.87	1.00
5	3	5.66	7.51	0.73	0.87	0.90	0.46	0.66	1.65	0.89
5	4	6.75	7.64	5.23	1.07	1.35	0.77	0.45	1.78	0.75
5	5	7.70	8.72	7.12	0.88	1.02	0.61	0.48	1.53	1.02

[a] 5 individuals of adults were used for this experiment. The pictures were taken 7 days after treatment.

[b] The 5 photos of a part of each symbiotic organ in an adult bean bug were taken by laser confocal microscope.

[c] The area means mm^2. ImageJ software (https://imagej.net/ij/) measured the DTN area (mm^2/insect) with a total area of 5 pictures.

[d] Each abbreviation means the following: DW, Distilled water; NAC, *N* -Acetyl-L-cysteine; L-Cys, L-cysteine; L-CME, L-cysteine methyl ester hydrochloride; 2-AET, 2-amino ethanethiol; L-Met, L-methionine; L-MME, L-methionine methyl ester hydrochloride.
